# Impaired Angiogenic Function of Fetal Endothelial Progenitor Cells via *PCDH10* in Gestational Diabetes Mellitus

**DOI:** 10.3390/ijms242216082

**Published:** 2023-11-08

**Authors:** Hayan Kwon, Yun Ji Jung, Yeji Lee, Ga-Hyun Son, Hyun Ok Kim, Yong-Sun Maeng, Ja-Young Kwon

**Affiliations:** 1 Department of Obstetrics and Gynecology, Institute of Women’s Life Medical Science, Yonsei University College of Medicine, Seoul 03722, Republic of Korea; whitekwon@yuhs.ac (H.K.); ccstty@yuhs.ac (Y.J.J.); herb1006@yuhs.ac (Y.L.); 2 Department of Obstetrics and Gynecology, Kangnam Sacred Heart Hospital, Hallym University Medical Center, Hallym University College of Medicine, Seoul 07441, Republic of Korea; ntr5017@naver.com; 3 Korea Cell-Based Artificial Blood Project, Regenerative Medicine Acceleration Foundation, Seoul 04512, Republic of Korea; hyunok1019@yuhs.ac

**Keywords:** gestational diabetes mellitus, endothelial progenitor cells, angiogenesis, *PCDH10*, epigenetic changes

## Abstract

Maternal hyperglycemia, induced by gestational diabetes mellitus (GDM), has detrimental effects on fetal vascular development, ultimately increasing the risk of cardiovascular diseases in offspring. The potential underlying mechanisms through which these complications occur are due to functional impairment and epigenetic changes in fetal endothelial progenitor cells (EPCs), which remain less defined. We confirm that intrauterine hyperglycemia leads to the impaired angiogenic function of fetal EPCs, as observed through functional assays of outgrowth endothelial cells (OECs) derived from fetal EPCs of GDM pregnancies (GDM-EPCs). Notably, *PCDH10* expression is increased in OECs derived from GDM-EPCs, which is associated with the inhibition of angiogenic function in fetal EPCs. Additionally, increased *PCDH10* expression is correlated with the hypomethylation of the *PCDH10* promoter. Our findings demonstrate that in utero exposure to GDM can induce angiogenic dysfunction in fetal EPCs through altered gene expression and epigenetic changes, consequently increasing the susceptibility to cardiovascular diseases in the offspring of GDM mothers.

## 1. Introduction

Gestational diabetes mellitus (GDM) is defined as a glucose intolerance resulting in maternal hyperglycemia, with onset or first recognition occurring during pregnancy. GDM complicates 5–31.5% of all pregnancies, and its prevalence has been rapidly increasing around the world alongside the rises in obesity and maternal age among women of reproductive age and advanced maternal age [1,2]. GDM is of great concern for fetal development and offspring health, as it is associated with adverse long-term outcomes in offspring as well as maternal and perinatal morbidity. Offspring that are exposed to GDM have an increased susceptibility to chronic diseases such as metabolic syndrome, type 2 diabetes, and hypertension, which include a cluster of cardiovascular diseases (CVDs) [3,4,5,6,7].

Although the pathogenesis linking intrauterine exposure to GDM with CVD in offspring is unknown, evidence suggests that an intrauterine hyperglycemic environment induces persistent epigenetic changes resulting in functional alterations in offspring. Several studies have shown that the DNA methylation of placental genes changes in GDM pregnancies, which is related to the regulation of adipose tissues, lipid transport, and inflammation in offspring [8,9]. Another study reported that hyperglycemic conditions significantly disrupt the development of the definitive endoderm, which is involved in the early stages of the development of the pancreas via histone H3 methylation [10]. Floris et al. demonstrated that alterations in micro-RNA expression in fetal endothelial cells from the umbilical cord vein of GDM pregnancies involved a complex in the initiation and maintenance of histone H3 methylation [11]. These findings suggest that in utero exposure to GDM can program epigenetic changes during fetal development, which may have long-lasting effects on gene expression and functional alterations in various tissues, leading to an increased risk of metabolic complications and CVD in offspring in later life.

The increased cardiovascular risk in offspring exposed to GDM could be related to endothelial progenitor cells (EPCs). EPCs, differentiated into endothelial cells, play a key role in both endothelial repair and neovascularization [12,13]. Previous studies have demonstrated that hyperglycemia is associated with reducing the number and angiogenic activity of EPCs significantly, resulting in the further progression of vascular disease [14,15,16,17]. During pregnancy, fetal exposure to a diabetic intrauterine environment results in decreased numbers and aberrant functions in cord blood-derived EPCs, which can be considered as fetal EPCs [10,11,18]. Fetal EPCs are believed to play a crucial role in the formation of fetal vasculature and the maintenance of vascular integrity. Therefore, the dysfunction of fetal EPCs could reflect the damage of stem cells, and may lead to vascular dysfunction in offspring in later life. However, whether and how intrauterine hyperglycemia induced by GDM contributes to fetal EPC dysfunction has remained unclear.

Based on previous studies, we hypothesized that intrauterine hyperglycemia exposure in the offspring of GDM mothers prompts the onset of endothelial dysfunction by altering the function of fetal EPCs through epigenetic changes, ultimately affecting the health of offspring. We aimed to investigate the programming effects of GDM on fetal EPCs and elucidate the underlying mechanisms related to adverse outcomes in offspring. We evaluate the angiogenic function of fetal EPCs exposed to GDM and evaluate related genes with epigenetic modification.

## 2. Results

### 2.1. GDM Reduced the Endothelial Angiogenic Capacity of Fetal EPCs

To investigate the effects of GDM on fetal EPCs, we differentiated cord blood-derived EPCs into outgrowth endothelial cells (OECs) and performed cell migration, adhesion, tube formation, and proliferation assays to assess the angiogenic capacity.

**Figure 1:**
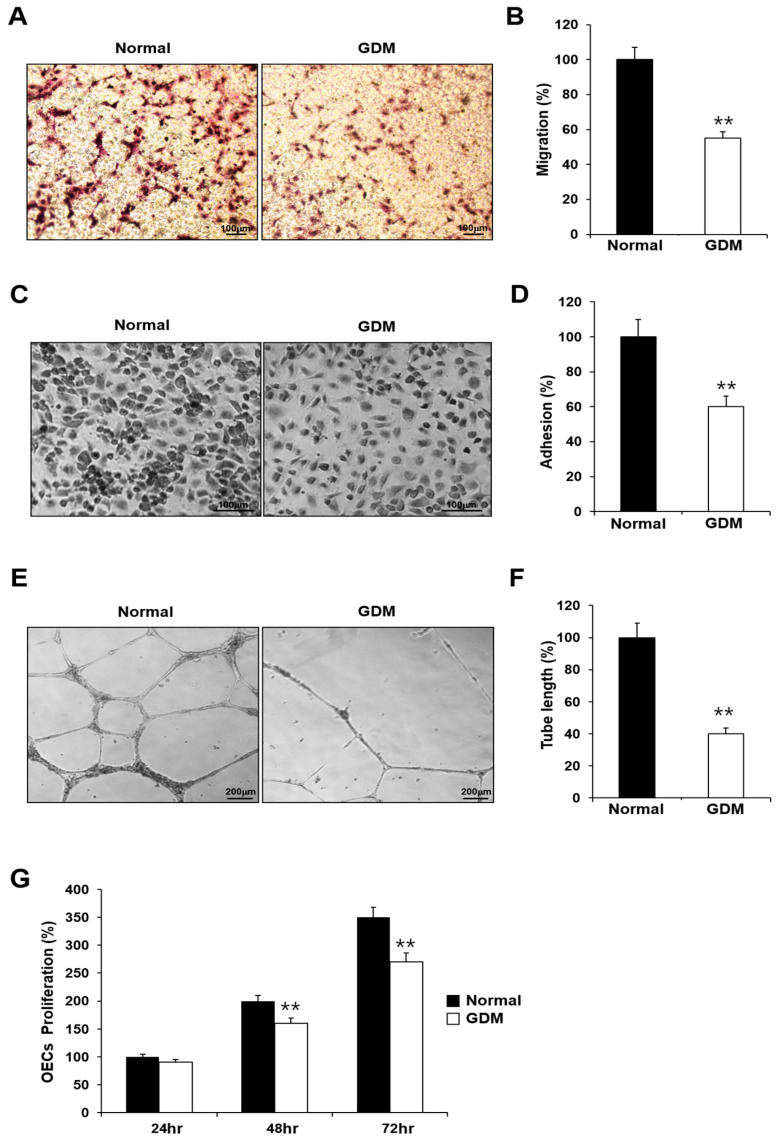
GDM impaired the endothelial angiogenic capacity in fetal EPCs. (**A**,**B**) Cell migration assay using a Transwell chamber after culture for 4 h, showing representative images of the H&E staining (**A**) and quantification (**B**). (**C**,**D**) Cell–matrix adhesion assay after incubation for 30 min, showing representative images of H&E staining (**C**) and quantification (**D**). (**E**,**F**) Tube formation assay following cell incubation on Matrigel for 18–24 h, showing representative images (**E**) and quantification (**F**). (**G**) Proliferation assay using MTT; the absorbance value (OD) of each well was measured at 490 nm at 24 h, 48 h, and 72 h. All data are presented as the mean ± SE. ** *p* < 0.01. *n* = 3 independent experiments for each assay.

Although the EPCs from GDM mothers (GDM-EPCs) exhibited a similar differentiation activity in terms of both the number and time for differentiation into OECs when compared to EPCs from the normal group (N-EPCs), the OECs differentiated from GDM-EPCs showed a significantly reduced migration capacity, decreased adherence to fibronectin, diminished tube formation, and reduced proliferative activity (Figure 1). These findings indicated that even fetal EPCs exposed to GDM can differentiate to OECs as under normal conditions, but the angiogenic capacity of OECs, including their migration, adhesion, tube formation, and proliferation, is impaired by in utero exposure to GDM.

### 2.2. Exposure to High-Glucose Conditions Impaired the Functional Capacity of Fetal EPCs

**Figure 2:**
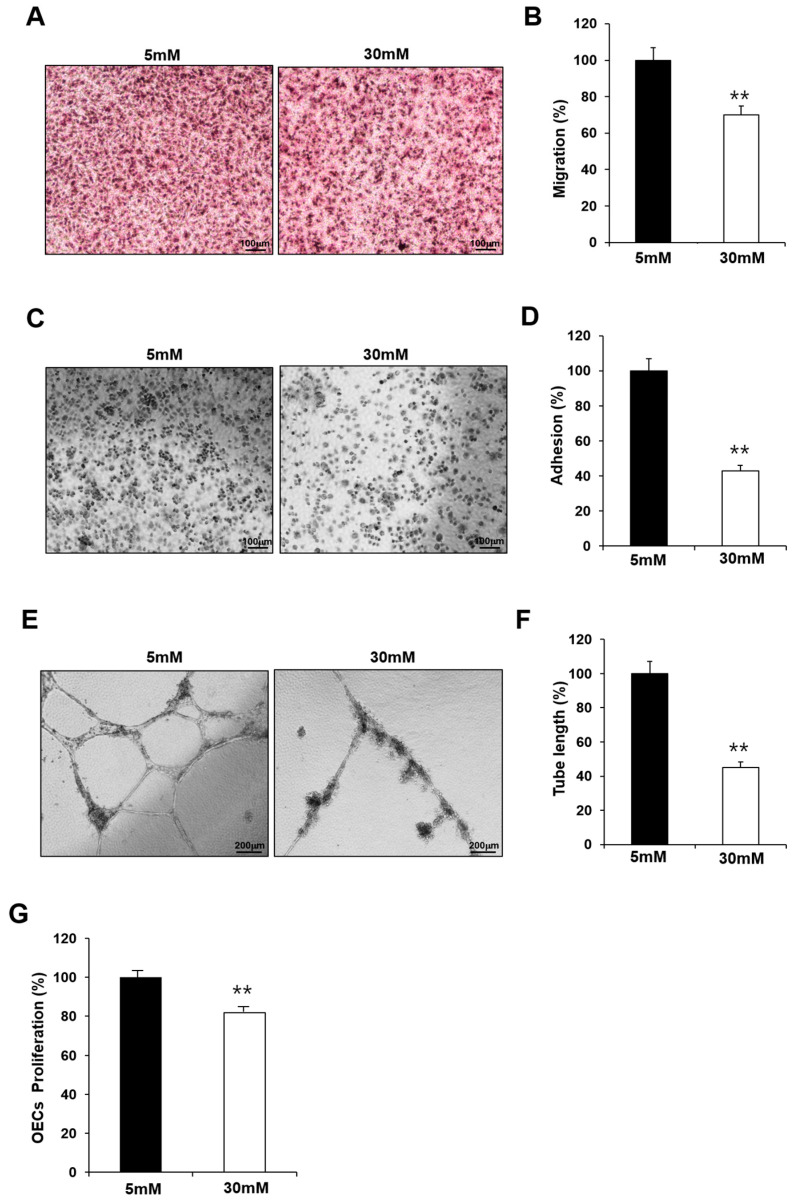
Exposure to high-glucose conditions reduced the angiogenic capacity in fetal EPCs, similar to that in cells from GDM. N-EPCs were exposed to normal D-glucose (5 mM) or high D-glucose (30 mM) concentrations. (**A**,**B**) Cell migration assay using a Transwell chamber after culture for 4 h, showing representative images of H&E staining (**A**) and quantification (**B**). (**C**,**D**) Cell–matrix adhesion assay after incubation for 30 min, showing representative images of H&E staining (**C**) and quantification (**D**). (**E**,**F**) Tube formation assay following cell incubation on Matrigel for 18–24 h, showing representative images (**E**) and quantification (**F**). (**G**) Proliferation assay using MTT; the absorbance value (OD) of each well was measured at 490 nm at 72 h. All data are presented as the mean ± SE. ** *p* < 0.01. *n* = 3 independent experiments for each assay.

GDM is associated with endothelial dysfunction, and maternal hyperglycemia is considered an independent predictor of childhood vascular complications. To confirm the effect of hyperglycemia, we examined whether the observed impairment in the fetal EPCs exposed to GDM was reproduced in N-EPCs in high-glucose conditions. For this purpose, N-EPCs were cultured in high-glucose conditions and were differentiated into OECs. Like the GDM-EPCs, the OECs that differentiated under high-glucose conditions had a decreased capacity for cell migration, adhesion, tube formation, and proliferation when compared with those that differentiated under normal conditions (Figure 2). These results indicate that fetal EPC dysfunction is associated with the high-glucose milieu induced by GDM.

### 2.3. PCDH10 Gene Expression Was Significantly Upregulated in GDM-EPCs as Well as in N-EPCs under Hyperglycemic Conditions

**Figure 3:**
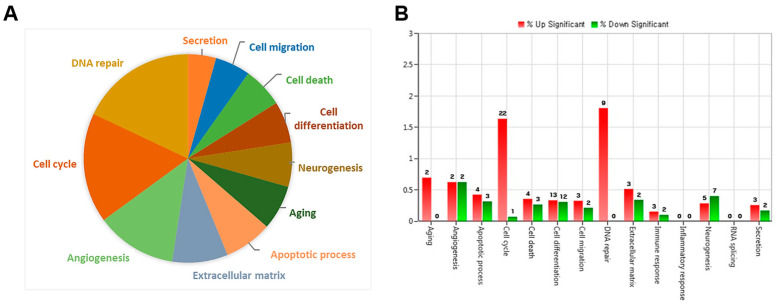
Gene expression patterns of OECs from GDM-EPCs compared with those from N-EPCs. (**A**) Pie charts show the transcripts significantly up-regulated and down-regulated in OECs from GDM-EPCs relative to the normal pregnancy. (**B**) Gene ontology analyses included genes that showed a *p*-value < 0.05 and a fold change > 2.0 and were performed using DAVID Bioinformatics Resources.

**Figure 4:**
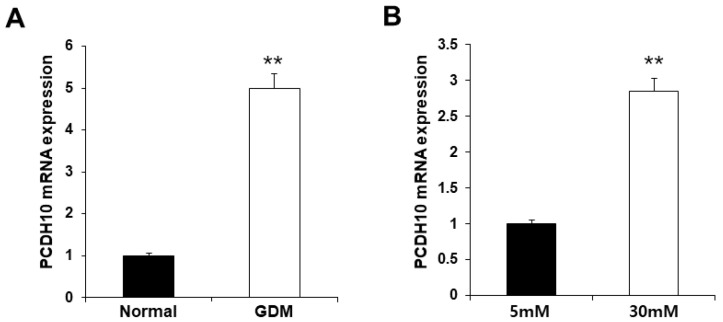
Significantly increased *PCDH10* expression in OECs from GDM-EPCs and in OECs from N-EPCs exposed to high-glucose conditions. (**A**) *PCDH10* expression using qRT-PCR in OECs derived from N-EPCs and GDM-EPCs. (**B**) *PCDH10* expression using qRT-PCR in OECs from N-EPCs exposed to normal glucose (5 mM) and high-glucose conditions (30 mM). Data are shown as the mean ± SE from three independent experiments. ** *p* < 0.01. NL, normal pregnancy; GDM, gestational diabetes mellitus; NG, normal glucose.

To investigate the molecular mechanisms underlying endothelial dysfunction in GDM and high-glucose conditions, the gene expression analysis of OECs from GDM-EPCs was compared with those from normal pregnancies (Figure 3). The profiles identified 107 genes whose expression was up-regulated > 2.0-fold (with *p* < 0.05) and 57 genes whose expression was down-regulated < 0.5-fold (with *p* < 0.05) in the OECs derived from GDM-EPCs. The up-regulated genes belong to a diverse set of categories, including the cell cycle, apoptotic process, extracellular matrix, and secretion. In contrast, down-regulated genes were related to angiogenesis, neurogenesis, cell differentiation, and immune response. A functional gene with potential relevance to endothelial dysfunction in GDM was then selected. Among the significantly up- and down-regulated genes, *PCDH10* was of particular interest because of the coincidence between the gene expression profiles and the qRT-PCR analysis of gene expression, the correlation of the effect on angiogenic function with our results, its possible association with epigenetic modification, and the consideration of a higher fold change of 6.35 and lower *p*-values (Table 1). *PCDH10* expression appeared to be significantly up-regulated in the OECs derived from GDM-EPCs compared to those derived from N-EPCs, and also in OECs from N-EPCs cultured under hyperglycemic conditions compared to those from N-EPCs and N-EPCs under normal conditions (Figure 4).

### 2.4. Knockdown of PCDH10 Recovered the Deteriorated Angiogenic Functions in GDM-EPCs

**Figure 5:**
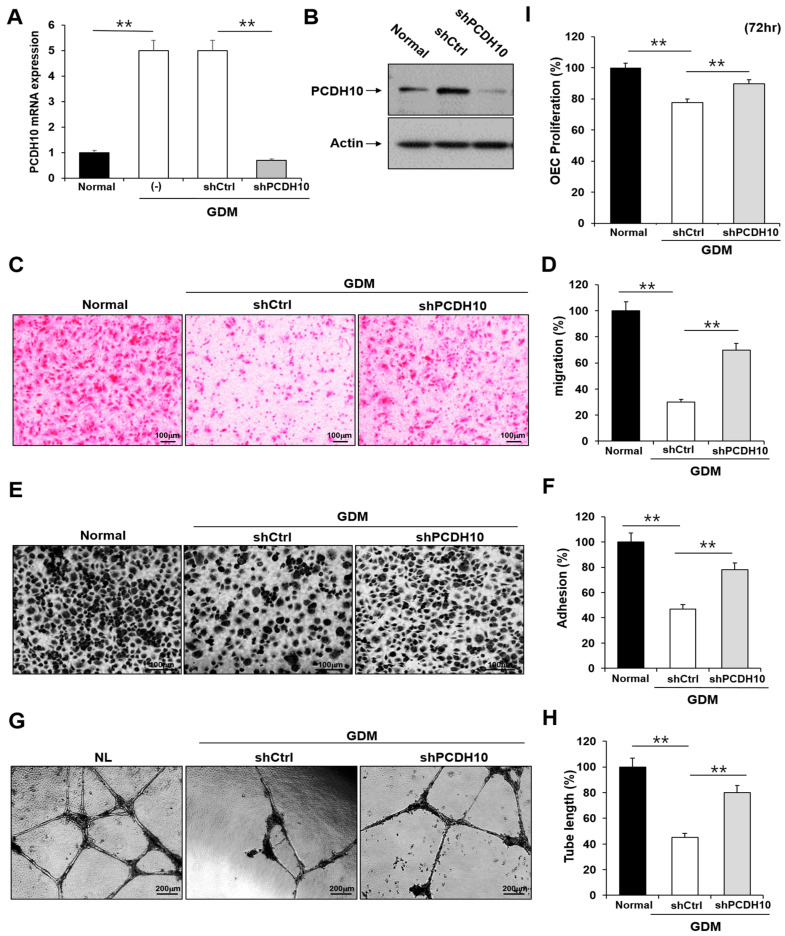
Knockdown of *PCDH10* expression in OECs from GDM-EPCs reversed the suppression of angiogenesis. (**A**) Knockdown efficiency using qRT-PCR in OECs derived from GDM-EPCs after transfection with sh*PCDH10*. (**B**) Representative image of Western blots of sh*PCDH10*-transfected OECs from GDM-EPCs. (**C**,**D**) Cell migration assay using a Transwell chamber after culture for 4 h, showing representative images of H&E staining (**C**) and quantification (**D**). (**E**,**F**) Cell–matrix adhesion assay after incubation for 30 min, showing representative images of H&E staining (**E**) and quantification (**F**). (**G**,**H**) Tube formation assay following cell incubation on Matrigel for 18–24 h, showing representative images (**G**) and quantification (**H**). (**I**) Proliferation assay using MTT; the absorbance value (OD) of each well was measured at 490 nm at 72 h. All data are presented as the mean ± SE. ** *p* < 0.01, comparison was performed between normal and GDM groups, and between the shCtrl- and sh*PCDH10*-transfected groups.

To determine whether *PCDH10* is involved in angiogenesis and the inhibitory effect on angiogenic function in EPCs, we examined the effect of *PCDH10* knockdown on the cell migration, adhesion, tube formation, and proliferation of OECs from GDM-EPCs (Figure 5). Lentivirus-mediated shRNA was used for the stable knockdown of *PCDH10* expression. The OECs from GDM-EPCs were infected with *PCDH10*-shRNA (sh*PCDH10*) and control shRNA (shCtrl) lentiviral particles. *PCDH10* expression was significantly reduced in the sh*PCDH10* group compared to control group in OECs from GDM-EPCs (Figure 5A,B). Functional assays were then conducted on the OECs from each group to assess cell migration, adhesion, tube formation, and proliferation (Figure 5C–I). The results revealed that *PCDH10* knockdown remarkably recovered the decreased capacity of cell migration and adhesion as well as tube formation in the GDM-OECs. Cell proliferation was also slightly increased in the *PCDH10*-knockdown group when compared with the decrease in the shCtrl group in the proliferation assay after 72 h. These results indicated that the increased expression of *PCDH10* in GDM-OECs significantly inhibits the migration, adhesion, tube formation, and proliferation of OECs, and that these effects are recovered through *PCDH10* knockdown.

### 2.5. High Glucose Induced Increased PCDH10 Expression in Fetal EPCs, Which Is an Irreversible Change That Did Not Revert to the Normal Levels Even under Normal Glycemic Conditions

**Figure 6:**
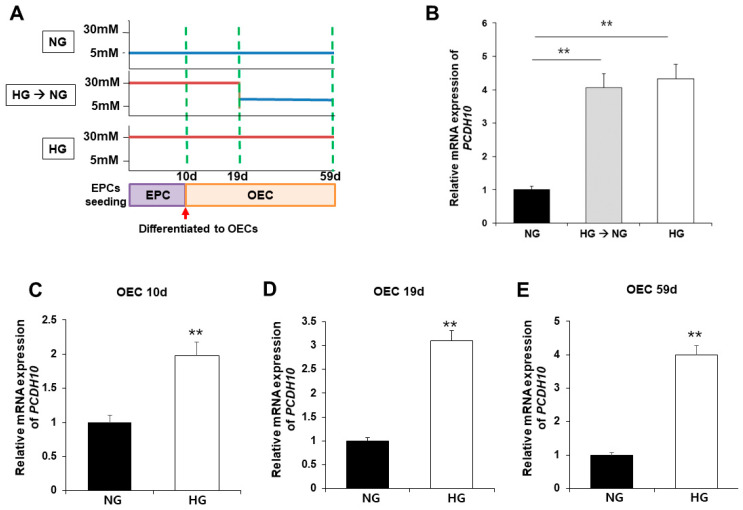
Exposure to high-glucose conditions increased *PCDH10* expression in OECs from N-EPCs, and the increased expression of *PCDH10* was not reversed under normal glucose conditions. (**A**) Schematic diagram of the procedure to evaluate the association of PCDH 10 expression and glucose conditions. (**B**) The expression of *PCDH10* in OECs under high-glucose conditions for 19 d followed by normal glucose conditions. High-glucose-induced increased *PCDH10* expression occurred even in the early passages of OECs, and the changes were not reversed even after return to normal conditions. (**C**–**E**) The expression of *PCDH10* in OECs derived from N-EPCs exposed to high-glucose conditions for 10 d, 19 d, and 59 d. ** *p* < 0.01. NG, normal glucose; HG, high glucose.

To evaluate the association of *PCDH10* expression with high glucose, *PCDH10* mRNA expression was assessed using qPCR and compared between OECs from N-EPCs cultured under normoglycemic (5 mM glucose) and hyperglycemic (30 mM glucose) conditions (Figure 6A). The results showed that, under high-glucose conditions, *PCDH10* mRNA expression levels were significantly increased. To determine the critical time for increased *PCDH10* expression, N-EPCs exposed to high-glucose conditions were categorized into three groups based on the duration of high glucose exposure: (1) OECs collected immediately after differentiation from EPCs (OEC 10 d; Figure 6C); (2) OECs under 30 mm glucose incubation for 19 days (OEC 19 d; Figure 6D); (3) OECs under 30 mm glucose incubation for 59 days (OEC 59 d; Figure 6E). The results showed that *PCDH10* was significantly up-regulated in all OECs cultured under high-glucose conditions (OEC 10 d, 19 d, 59 d). To investigate whether the increased expression caused by high glucose could return to baseline levels following exposure to a normal glucose environment, OECs under high-glucose conditions for 19 days were cultured at two different concentrations: normoglycemic (5 mM) and hyperglycemic (30 mM) conditions. As a result, increased *PCDH10* expression induced by high glucose was not significantly decreased, even under normal glycemic conditions (Figure 6B). These results showed that a high-glucose environment could induced increased *PCDH10* expression in fetal EPCs irreversibly, which was not rectified by the correction of the serum environment, indicating the possibility of an epigenetic modification. The results suggest that intrauterine hyperglycemia during critical periods of fetal vascular development results in deleterious effects on vascular health, which can persistently affect the health of the offspring later in life.

### 2.6. CpG Islands of the PCDH10 Promoter Was Significantly Hypomethylated in GDM-EPCs and N-EPCs under Hyperglycemic Conditions

**Figure 7:**
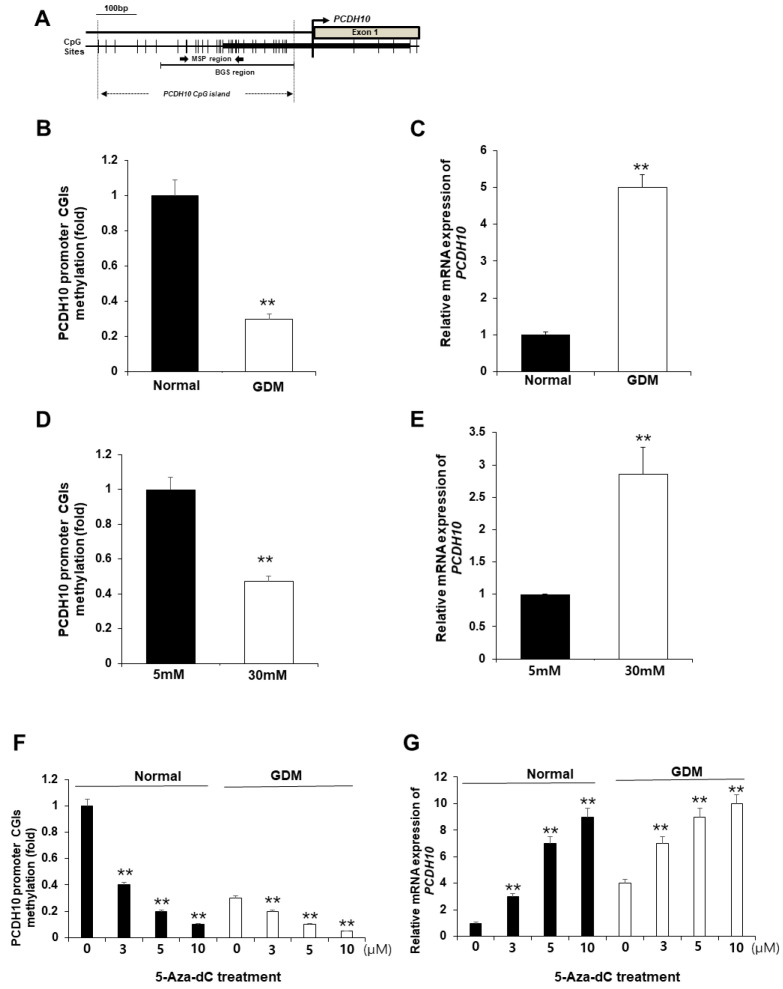
The promoter CGIs of *PCDH10* were hypomethylated in OECs from GDM-EPCs and N-EPCs exposed to high-glucose conditions, and correlated with increased *PCDH10* expression. (**A**) Schematic diagram of the CpG island of the *PCDH10* promoter. (**B**) Methylation status of *PCDH10* in OECs derived from GDM. (**C**) Relative mRNA expression of *PCDH10* in OECs derived from GDM. (**D**) Methylation status of *PCDH10* in OECs derived from N-EPCs exposed to normal and high-glucose conditions. (**E**) Relative mRNA expression of *PCDH10* in OECs derived from N-EPCs under normal glucose and high-glucose conditions. (**F**,**G**) Treatment with 5-Aza-dC induced hypomethylation of the *PCDH10* promoter CGIs with concomitant increased *PCDH10* mRNA expression in a dose-dependent manner. ** *p* < 0.01. NL, normal pregnancy; GDM, gestational diabetes mellitus; NG, normal glucose.

We hypothesized that the alterations in *PCDH10* mRNA expression in OECs derived from GDM-EPCs may be caused by epigenetic modification, specifically aberrant promoter methylation, which could contribute to vascular dysfunction in offspring. We assessed the methylation status of CpG islands (CGIs) in the *PCDH10* promoter (Figure 7A) using methylation-specific PCR (MSP) in both OECs derived from GDM-EPCs and OECs from N-EPCs cultured under high-glucose conditions, where we observed significantly increased *PCDH10* expression (Figure 7B–E). As expected, the CGIs was significantly hypomethylated in OECs derived from GDM-EPCs (Figure 7B) and OECs from N-EPCs cultured under high-glucose conditions (Figure 7D). These results suggest that GDM and exposure to hyperglycemic conditions might be involved in the methylation of the *PCDH10* promoter in fetal EPCs.

### 2.7. Pharmacologic Demethylation Activated PCDH10 Expression

To evaluate whether the hypomethylation of *PCDH10* promoter CGIs directly mediates increased *PCDH10* expression, OECs derived from N-EPCs were treated with the DNA methyltransferase inhibitor 5-Aza-dC, and *PCDH10* expression levels were compared before and after treatment. An MSP analysis showed that the *PCDH10* promoter CGIs were dramatically demethylated in the presence of the drug. *PCDH10* mRNA expression was significantly increased after treatment with 5-Aza-dC in a dose-dependent manner in OECs derived from EPCs of both normal and GDM pregnancies (Figure 7F,G). Based on these results, we confirmed that the expression of *PCDH10* is directly mediated by the methylation status of CGIs in fetal EPCs, indicating that epigenetic modifications are involved in *PCDH10* expression.

## 3. Discussion

Intrauterine exposure to hyperglycemia in GDM pregnancies is known to have an impact on long-term cardiovascular health in offspring [7,19]. Accumulating evidence suggests that these changes are triggered by epigenetic modifications in fetal cells during fetal developments [19,20,21,22,23]. During fetal developments, fetal EPCs play a potentially important role in embryonic neovascularization and support the health of the vascular endothelium. Epigenetic modifications have been proposed as potential factors linking intrauterine hyperglycemia exposure to later health outcomes; however, there have been limited studies investigating the functions of fetal EPCs in human offspring born to mothers with GDM pregnancies [24].

Our studies showed that the exposure of fetal EPCs to GDM in vivo or in hyperglycemic conditions in vitro could impact both the angiogenic function and gene expression of fetal EPCs. We demonstrated that *PCDH10* was significantly up-regulated in GDM, and that an increased expression of *PCDH10* was involved in endothelial dysfunction. Furthermore, it is particularly noteworthy that a reduction in *PCDH10* promoter methylation is linked to the expression of *PCDH10* in GDM and hyperglycemic conditions. Our study has a significant implication that we have found the novel mechanism mediating EPC dysfunction in intrauterine hyperglycemia induced by GDM. This observation also suggests that hyperglycemia in GDM influences the epigenetic modification of EPCs.

EPCs can promote angiogenesis and differentiate into mature endothelial cells. In order for angiogenesis to be achieved, endothelial cells must be activated, proliferated, and well adhered to the extracellular matrix alongside tube formations. Therefore, the function of EPCs may be essential for maintaining a healthy cardiovascular system. Although little is known about the effects of maternal GDM on fetal EPCs, a few studies suggest that GDM exposure in utero contributes to impaired function and leads to altered gene expression in fetal EPCs. Gui et al. reported that umbilical cord blood-derived endothelial colony-forming cells (ECFCs), also known as late EPCs or OECs, have significantly impaired angiogenic functions in the diabetic intrauterine environment when compared to those from heathy pregnancies. They also demonstrated the decreased expression and activity of SIRTs in fetal ECFCs and human umbilical vein endothelial cells from GDM pregnancies, which may be associated with long-term cardiovascular complications in offspring of GDM pregnancies [24,25]. Varerg KM et al. reported that GDM-exposed ECFCs have decreased vasculogenic potential and altered gene expression with vasculogenic dysfunction [26,27]. An increased expression of TAGLN involved in actin cytoskeletal rearrangement was associated with impaired ECFC migration, cell alignment, and network formation [28].

In accordance, our study demonstrated that OECs differentiated from EPCs exposed to GDM pregnancies or high-glucose environments resulted in marked decreases in angiogenesis when compared to those from EPCs with normal pregnancies or normoglycemic environments. This indicates that exposure to a hyperglycemic environment in utero has a significant impact on fetal EPCs, leading to functional impairments in OECs. It is reasonable to suppose that the intrauterine hyperglycemic environment during critical periods of fetal vascular development alters the ability of EPCs.

To identify possible molecular mechanisms contributing to the functional differences between normal and GDM-exposed EPCs, we conducted a gene expression analysis using mRNA sequencing. OECs differentiated from GDM-exposed EPCs exhibited modest differences to a control, as well as significantly up-regulated genes such as ROBO1, IGFBP1, and *PCDH10*, and down-regulated genes such as HEY1 were known to be related to angiogenesis in endothelial cells. Several studies have reported that Slit/Robo signaling with ROBO1 is involved in angiogenesis. Slit2/Robo1 binding exerts a pro-migratory effect on HUVECs, in which Robo4 provides an inhibitory effect against migration [29,30,31]. In a diabetic environment, IGFBP1 improves endothelial regeneration and restores endothelial reparative function through preserved insulin sensitivity and increased nitric oxide production [32,33]. In addition, the Hey family are direct targets of Notch signaling and Hey1/Hey2 are essential transducers of Notch signals in cardiovascular development [34,35]. Although these genes are related to hyperglycemia and angiogenesis in endothelial cells, *PCDH10* was of particular interest because of the correlation of the effect on angiogenic function with our results, as well as its possible association with epigenetic modification. We have demonstrated a novel underlying mechanism involving *PCDH10* as one of the genes that is significantly differently expressed in fetal OECs from GDM-exposed EPCs.

*PCDH10* belongs to the major subfamily of the cadherin superfamily and is involved in the establishment and function of cell-to-cell adhesion. Although the functions of protocadherins have not been well elucidated, recent studies suggest that *PCDH10* expression was frequently down-regulated in various cancers [36]. It has been reported that *PCDH10* acts as a functional tumor suppressor gene by inhibiting cell proliferation, migration, and clonogenicity, as well as through the induction of apoptosis [37,38]. In our study, we identified that increased *PCDH10* expression is involved in angiogenesis impairment in OECs from GDM-EPCs. The knockdown of *PCDH10* resulted in the remarkable restoration of cell adhesion, migration, tube formation, and proliferation, indicating its crucial role in angiogenesis. Furthermore, high-glucose environments contribute to the increased expression of *PCDH10* in OECs derived from normal fetal EPCs. Significantly increased *PCDH10* expression was observed not only in OECs exposed to high-glucose conditions for a long time after differentiation from EPCs, but also in OECs identified immediately after differentiation that were exposed to high-glucose conditions while only in the state of EPCs. These results indicated that transient high glucose exposure in utero can directly affect stem cells and alter the function of EPCs by altering *PCDH10* expression, which is related with vascular dysfunction.

Maternal glucose intolerance from GDM is recovered after childbirth, but in offspring, GDM increases the risk of CVD and metabolic disease, which can be explained by the fetal programming phenomenon. This is because maternal hyperglycemia provokes permanent epigenetic changes in gene expression. Our study shows that high glucose leads to altered gene expression and disrupted epigenetic regulation. *PCDH10* expression, which was increased in high-glucose conditions, was not restored even after incubation in normal glucose conditions. Increased *PCDH10* expression was still observed in OECs cultured after exposure to high-glucose conditions and then normal conditions. These significant findings provide the possibility that hyperglycemia during pregnancy contributes to irreversible changes to fetal stem cells, with an epigenetic modification that has long-lasting adverse effects on the vascular systems in the offspring of GDM mothers. These results also provide evidence for the importance of maternal glucose control in GDM and *PCDH10* expression to improve the health of offspring born to mothers with GDM.

Increasing evidence suggests that epigenetic modifications via DNA methylation in GDM affects the progression of diabetes-related vascular disease in offspring [39,40,41,42,43]. DNA methylation regulates gene expression and genomic stability. In previous studies, *PCDH10* was reported to be silenced via promoter methylation in various types of cancer [44,45,46,47]. Particularly, several studies demonstrated that *PCDH10* plays a positive role in angiogenesis, and that its expression is controlled via promoter methylation. In our study, hypomethylation was found to be negatively correlated with *PCDH10* mRNA expression in OECs from GDM-EPCs and OECs derived from N-EPCs exposed to high-glucose conditions. These results indicate that promoter hypomethylation is the principal regulatory mechanism for the increased expression of *PCDH10* in fetal EPCs following GDM or high glucose exposure, and finally leads to aberrant vascular health in the offspring. In addition, further cohort studies are needed to confirm whether these changes continue to imprint on offspring later in life, ultimately affecting their vascular health. Furthermore, based on the gene expression analysis of OECs from GDM-EPCs, further studies can be considered in genes related to angiogenesis, including ROBO1, IGFBP1, and PCDH7.

In conclusion, we demonstrated that fetal exposure to maternal GDM in utero can induce epigenetic modification and alter gene expression in fetal EPCs, resulting in the angiogenic dysfunction of fetal EPCs. It can be inferred that such an impact on epigenetic programming during fetal life predisposes offspring that are exposed to maternal GDM to developing cardiovascular diseases later in life. In addition, since epigenetic changes can occur in offspring exposed to GDM, this study also shows the importance of glucose control during pregnancy, and furthermore, it may suggest a target therapy and treatment to restore vascular health in patients diagnosed with DM or glucose intolerance.

## 4. Materials and Methods

### 4.1. Study Population and Sample Collection

Women with normal (*n* = 30) and GDM pregnancies (*n* = 30) were recruited from January 2018 to June 2021. All women assigned to the GDM group were diagnosed with two of the following: a fasting blood glucose > 5.3 mmol/L, a 1 h oral glucose tolerance test (OGTT) > 10.0 mmol/L, a 2 h OGTT > 8.6 mmol/L, or a 3 h OGTT > 7.8 mmol/L in 100 g OGTT followed by a 50 g glucose tolerance test > 7.5 mmol/L during 24–28 gestational weeks. Cases of preterm delivery < 36 gestational weeks, multiple pregnancies, pregnancies associated with fetal malformation, chromosome anomalies, diabetes diagnosed before pregnancy, chronic hypertension or preeclampsia, and extreme maternal obesity (pre-pregnancy body mass index ≥ 30 kg/m^2^) were excluded from this study. To determine the effects of intrauterine hyperglycemia on the fetus, our study investigated various cases of pregnant women with poor glycemic control for approximately 9–10 weeks from GDM diagnosis to delivery. Because the fetal circulatory system is connected through the umbilical cord to the mother’s blood, and umbilical cord blood can reflect the fetal condition, umbilical cord blood for fetal EPCs (CD133^+^/C-kit^+^/Lin^−^ cells: CKL- cells) was obtained at the time of delivery.

### 4.2. Function Evaluation of Fetal EPCs from the Umbilical Cord Blood 

#### 4.2.1. Isolation and Culture of Fetal EPCs (CKL- Cells)

**Figure A1:**
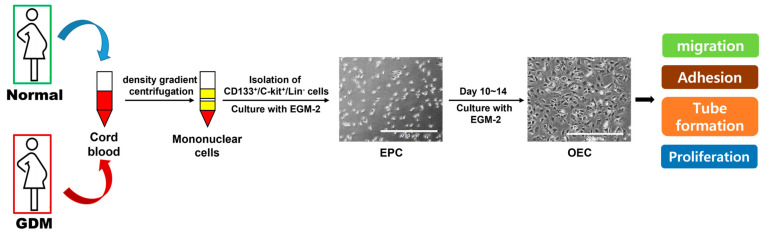
Schematic image of the differentiation of EPCs to OECs.

Fetal EPCs were prepared as previously described [48]. Briefly, cord blood samples containing fetal EPCs were collected prior to placental expulsion via gravity flow at the time of delivery. EPCs were isolated using density gradient centrifugation at 400× *g* for 30 min using Biocoll (Biochrom, Berlin, Germany) and washed three times in phosphate-buffered saline (PBS; Biochrom). CKL- cells were purified via positive and negative selection with anti-CD133/C-kit/Lin− microbeads (Miltenyi Biotec, Bergisch-Gladbach, Germany) using a magnetic cell sorter device (Miltenyi Biotec). The purity of the cell fraction was assessed using a fluorescence-activated cell sorting analysis and was confirmed as > 98%. The CKL- cells were seeded into 6-well plates coated with human fibronectin (Sigma, St. Louis, MO, USA) and maintained in endothelial basal medium 2 (EBM-2; Clonetics, Cell Systems, St. Katharinen, Germany). The medium was supplemented with endothelial growth medium 2 (EGM-2; Clonetics, Cell Systems) containing fetal bovine serum, human VEGF-A, human fibroblast growth factor-B, human epidermal growth factor-B, IGF1, and ascorbic acid. The identification of CKL- cells was determined via staining with human CD133 phycoerythrin (PE)-conjugated antibodies and C-kit PE-conjugated antibodies (BD Biosciences, Bedford, MA, USA). For further information, see Figure A1 in Appendix A.

#### 4.2.2. CKL- Cells Differentiation Assay 

EPCs from GDM (GDM-EPCs) and normal pregnancies (N-EPCs) were seeded on 6-well plates (1 × 10^6^ cells/well) and cultured in the EGM-2 medium, and the medium was replaced every 2 days. EPCs have the potential to differentiate to form outgrowth endothelial cells (OECs). The day of differentiation was defined as the first day on which a differentiated colony was observed from the time of seeding. The duration required for the differentiation of the colonies in each set to occur was determined using light microscopy. At least three assays were performed for each sample.

#### 4.2.3. Cell Migration Assay

Cell migration was assessed using Transwell^®^ Permeable Supports (Corning Costar, Acton, MA, USA) with a 6.5 mm diameter polycarbonate membrane (8.0-µm pore size). The lower surface of the membrane was coated with 10 μg/mL of fibronectin (Sigma-Aldrich Corp., St. Louis, MO, USA). OECs at passage 3 (105 cells) were seeded onto chemotaxis filters in the insert with EBM supplemented with 0.5% FBS. After 4 h of incubation at 37 °C, the insert was washed with PBS and non-migrating cells in the top surface of the membrane were removed. Migrating cells attached to the lower surface of the filters were stained with hematoxylin and eosin (H&E) and quantified using Kodak 1D software 3.6 (Eastman Kodak, Rochester, NY, USA). The assays were performed in triplicate with three different samples [49].

#### 4.2.4. Cell–Matrix Adhesion Assay

Cell–matrix adhesion assays were performed as previously described [49]. Briefly, 96-well plates were coated overnight at 4 °C with 10 µg/mL of human fibronectin (Sigma-Aldrich, St. Louis, MO, USA). At the 3rd passage, OECs in a 100 µL adhesion buffer comprising serum-free media and EBM were seeded at 105 cells/well and incubated for 30 min at 37 °C. After two washes with PBS to remove non-adherent cells, the remaining adherent cells were measured via H&E staining and quantified in triplicate by counting adherent cells in five randomly selected fields per well (Axiovert 100; Carl Zeiss Micro-Imaging, Thornwood, NY, USA). The assays were performed in triplicate with three different samples.

#### 4.2.5. Tube Formation Assay

A tube formation assay was conducted as previously described [49]. The Matrigel solution (250 µL; BD Biosciences, Bedford, MA, USA) was added to a 16 mm diameter tissue culture well and allowed to polymerize for 30 min at 37 °C. After trypsinization, the harvested OECs (1.2 × 10^5^ cells/well) were resuspended in EBM and plated onto the Matrigel. The Matrigel cultures were incubated at 37 °C, and the cells were photographed at 18 h and 24 h of incubation (200× magnification). The area covered by the mature tube network was determined by scanning photographs of the tubes into Adobe Photoshop 23.5.5 and using the ImageJ software (Version 1.53t 24 August 2022, National Institute of Health, Bethesda, MD, USA) to quantify the identified area and measure the total tube length on the captured images.

#### 4.2.6. Proliferation Assay

The OECs (1 × 10^3^ cells) were seeded onto a 96-well plate, cultured for 72 h, and then incubated with 20 µL of 3-(4,5-dimethylthiazol-2-yl)-2,5-diphenyl tetrazolium bromide (MTT; 5 mg/mL) reagent. After 4 h, the supernatants were removed, and the cells were treated with 150 μL of dimethyl sulfoxide (DMSO). The absorbance value (optical density, OD) at a wavelength of 490 nm was measured at 24 h, 48 h, and 72 h. The experiments were performed in triplicate with three different cell lines [49].

### 4.3. Analysis of the Gene Expression Profiles Using mRNA Sequencing

Total RNA was isolated using the TRIzol^TM^ Reagent (Life Technologies, Van Allen Way, CA, USA), and the RNA quality was assessed on an Agilent 2100 Bioanalyzer using the RNA 6000 Nano Chip (Agilent Technologies, Amstelveen, The Netherlands). RNA quantification was performed using an ND-2000 Spectrophotometer (Thermo Fisher Scientific Inc., Wyman Street Waltham, MA, USA).

For the normal (*n* = 3) and GDM (*n* = 3) pregnancy-derived OEC RNAs, library construction was performed using a QuantSeq 30-mRNA-Seq Library Prep Kit (Lexogen, Inc., Vienna, Austria) according to the manufacturer’s instructions [49]. In brief, 500 ng of total RNA was prepared, hybridized with an oligo-dT primer containing an Illumina-compatible sequence at its 5′-end, and subjected to reverse transcription. After the degradation of the RNA template, second-strand synthesis was initiated using a random primer containing an Illumina-compatible linker sequence at its 5′-end. The double-stranded library was purified using magnetic beads to remove all the reaction components. The library was amplified to add the complete adapter sequences required for cluster generation. The finished library was purified from PCR components, and high-throughput sequencing was performed via single-read sequencing (75 cycles) using NextSeq 500 (Illumina, Inc., San Diego, CA, USA). The QuantSeq 30-mRNA-Seq reads were aligned using Bowtie 2. The Bowtie 2 indices were generated from either the genome assembly sequence or the representative transcript sequence to align the genome and transcriptome. The alignment file was used to assemble the transcripts, estimate their abundances, and detect differential gene expression. Differentially expressed genes were determined based on the counts from unique and multiple alignments using coverage in BEDtools version 2.28.0. The read count (RC) data were processed based on the quantile normalization method using EdgeR (version 3.18) within the R program (version 4.3) in Bioconductor. Gene classification was based on searches performed using DAVID (https://david.ncifcrf.gov/, accessed on 17 May 2021) and the Medline database (http://www.ncbi.nlm.nih.gov/, accessed on 17 May 2021).

### 4.4. Total RNA Extraction, Reverse Transcription, and Quantitative Real-Time PCR (qRT-PCR)

Total RNA was extracted from the EPCs and OECs using the TRIzol^TM^ Reagent (Life Technologies, Van Allen Way, CA, USA). The mRNA expression of *PCDH10* was measured using a Power SYBR^TM^ Green RNA-to-CT^TM^ 1-Step kit (Applied Biosystems, Foster City, CA, USA) and StepOnePlus^TM^ (Applied Biosystems) according to the manufacturer’s instructions. GAPDH served as an internal standard for sample normalization. The conditions for amplification were as follows: 48 °C for 30 min and 95 °C for 10 min, followed by 40 cycles of 95 °C for 15 s and 55 °C for 1 min. The results were based on the cycle threshold (Ct) values. The relative gene expression levels were calculated using the comparative CT method (2^−△△Ct^) with GAPDH as an internal control. The assay for the expression of *PCDH10* via qRT-PCR used the following primers: *PCDH10* forward 5′- AGC TCC AAT GTA CCC AGT AA-3′, reverse 5′- CAG GGC TTA AGA AAC ATC AG-3′; GAPDH forward 5′-GGG GTC ATT GAT GGC AAC AA -3′, reverse 5′-ATG GGG AAG GTG AAG GTC G -3′.

### 4.5. PCDH10 shRNA Infection

**Figure A2:**
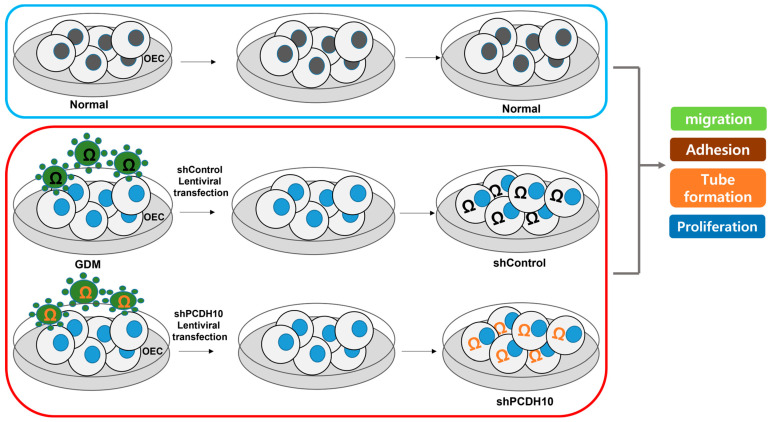
Lentiviral transfection workflow for *PCDH10* knockdown as described in the Section 4.

To knockdown the expression of *PCDH10*, OECs from GDM-derived EPCs were cultured in 6-well plates at a density of 5 × 10^4^ cells per well in 2 mL of EGM-2 and incubated for 24 h. They were maintained in 2 mL of a complete optimal medium (with serum and antibiotics) and incubated overnight until 60–70% confluence on the day of infection. The cells were then infected with shRNA lentiviral particles, which were constructed to target for *PCDH10* (sh*PCDH10*; Santa Cruz Biotechnology, Santa Cruz, CA, USA), with nontargeting sequence infection as a control (shCtrl), following the manufacturer’s instructions. Infected cells were selected using puromycin. The expression of *PCDH10* mRNA was analyzed using qRT-PCR after selection. For further information, see Figure A2 in Appendix A.

### 4.6. Exposure to High-Glucose Conditions

To evaluate the effects of maternal hyperglycemia on fetal EPCs, N-EPCs were cultured in high D-glucose (30 mM) and normal D-glucose (5 mM). Cells for functional assays were treated for 19 days. For the quantification of *PCDH10* expression, the cells were treated for 10 days, 19 days, and 59 days from the day of differentiation into OECs. To evaluate the expression changes in *PCDH10*, the N-EPCs were exposed to high-glucose conditions for 19 days after differentiation into OECs and then cultured in the presence of two different concentrations (5 mM and 30 mM) for 27 days. At least three assays were performed for each sample.

### 4.7. DNA Bisulfite and Methylation Analysis

DNA was extracted from OECs from normal and GDM pregnancies, as well as OECs exposed to high-glucose conditions. Bisulfite modifications of DNA and the methylation status in the CGIs of the *PCDH10* promoter was carried out as previously described. The bisulfite-treated DNA was amplified using the methylation-specific primer set, *PCDH10*-M1 5′-TCG TTA AAT AGA TAC GTT ACG C-3′, *PCDH10*-M2, 5′-TAA AAA CTA AAA ACT TTC CGC G-3′, or the unmethylation-specific primer set, *PCDH10*-U1, 5′-GTT GTT AAA TAG ATA TGT TAT GT-3′, *PCDH10*-U2, 5′-CTA AAA ACT AAA AAC TTT CCA CA-3′. Methylation-specific PCR (MSP) was performed for 40 cycles using the EpiScope MSP kit (Takara Bio Inc., Kusatsu, Japan).

### 4.8. DNA Demethylation Using the 5-Aza-2′-deoxycytidine (5-Aza-dC)

The OECs were treated with 3, 5, and 10 μM of the DNA demethylating agent 5-Aza-dC for 24 h. The cells were then harvested for DNA and RNA extraction.

### 4.9. Statistical Analysis

Data are presented as the mean ± standard error (SE). Statistical significance between groups were assessed using a one-way analysis of variance (ANOVA), followed by Student’s *t*-test. A *p* < 0.01 was considered statistically significant.

## Figures and Tables

**Table 1:** The fold change and *p*-value of *PCDH10*.

Gene Symbol	Fold Change	*p*-Value
*COL1A2*	19.420	0.009
*DCN*	11.877	0.000
*SNAR-A12*	11.429	0.038
*RIC3*	9.720	0.018
*SNAR-A8*	8.633	0.049
*SNAR-A3*	8.617	0.041
*SNHG5*	8.397	0.010
*HSPA2*	8.091	0.009
*TCEAL7*	7.945	0.001
*ROBO1*	6.708	0.007
*IGFBP1*	6.641	0.036
***PCDH10***	**6.352**	**0.012**
*AR*	5.745	0.013
*PCDH7*	5.432	0.016
*SCN8A*	5.426	0.031

## Data Availability

The data that support the finding of this study are available from the corresponding author upon reasonable request.

## References

[1] Metzger B.E., Buchanan T.A., Coustan D.R., de Leiva A., Dunger D.B., Hadden D.R., Hod M., Kitzmiller J.L., Kjos S.L., Oats J.N. (2007). Summary and recommendations of the fifth international workshop-conference on gestational diabetes mellitus. Diabetes Care.

[2] Zhu Y., Zhang C. (2016). Prevalence of gestational diabetes and risk of progression to type 2 diabetes: A global perspective. Curr. Diab. Rep..

[3] Wright C.S., Rifas-Shiman S.L., Rich-Edwards J.W., Taveras E.M., Gillman M.W., Oken E. (2009). Intrauterine exposure to gestational diabetes, child adiposity, and blood pressure. Am. J. Hypertens..

[4] Boney C.M., Verma A., Tucker R., Vohr B.R. (2005). Metabolic syndrome in childhood: Association with birth weight, maternal obesity, and gestational diabetes mellitus. Pediatrics.

[5] Bunt J.C., Tataranni P.A., Salbe A.D. (2005). Intrauterine exposure to diabetes is a determinant of hemoglobin a(1)c and systolic blood pressure in pima indian children. J. Clin. Endocrinol. Metab..

[6] Cho N.H., Silverman B.L., Rizzo T.A., Metzger B.E. (2000). Correlations between the intrauterine metabolic environment and blood pressure in adolescent offspring of diabetic mothers. J. Pediatr..

[7] Bianco M.E., Josefson J.L. (2019). Hyperglycemia during pregnancy and long-term offspring outcomes. Curr. Diab. Rep..

[8] Cardenas A., Gagne-Ouellet V., Allard C., Brisson D., Perron P., Bouchard L., Hivert M.F. (2018). Placental DNA methylation adaptation to maternal glycemic response in pregnancy. Diabetes.

[9] Houde A.A., St-Pierre J., Hivert M.F., Baillargeon J.P., Perron P., Gaudet D., Brisson D., Bouchard L. (2014). Placental lipoprotein lipase DNA methylation levels are associated with gestational diabetes mellitus and maternal and cord blood lipid profiles. J. Dev. Orig. Health Dis..

[10] Chen A.C.H., Lee Y.L., Fong S.W., Wong C.C.Y., Ng E.H.Y., Yeung W.S.B. (2017). Hyperglycemia impedes definitive endoderm differentiation of human embryonic stem cells by modulating histone methylation patterns. Cell Tissue Res..

[11] Floris I., Descamps B., Vardeu A., Mitic T., Posadino A.M., Shantikumar S., Sala-Newby G., Capobianco G., Mangialardi G., Howard L. (2015). Gestational diabetes mellitus impairs fetal endothelial cell functions through a mechanism involving microrna-101 and histone methyltransferase enhancer of zester homolog-2. Arter. Thromb. Vasc. Biol..

[12] Eguchi M., Masuda H., Asahara T. (2007). Endothelial progenitor cells for postnatal vasculogenesis. Clin. Exp. Nephrol..

[13] Salybekov A.A., Kobayashi S., Asahara T. (2022). Characterization of endothelial progenitor cell: Past, present, and future. Int. J. Mol. Sci..

[14] Altabas V. (2015). Diabetes, endothelial dysfunction, and vascular repair: What should a diabetologist keep his eye on?. Int. J. Endocrinol..

[15] Rigato M., Avogaro A., Fadini G.P. (2016). Levels of circulating progenitor cells, cardiovascular outcomes and death: A meta-analysis of prospective observational studies. Circ. Res..

[16] Rigato M., Bittante C., Albiero M., Avogaro A., Fadini G.P. (2015). Circulating progenitor cell count predicts microvascular outcomes in type 2 diabetic patients. J. Clin. Endocrinol. Metab..

[17] Wei H.-J., Liu L., Chen F.-L., Wang D., Wang L., Wang Z.-G., Jiang R.-C., Dong J.-F., Chen J.-L., Zhang J.-N. (2019). Decreased numbers of circulating endothelial progenitor cells are associated with hyperglycemia in patients with traumatic brain injury. Neural Regen. Res..

[18] Dincer U.D. (2015). Fetal exposure to a diabetic intrauterine environment resulted in a failure of cord blood endothelial progenitor cell adaptation against chronic hypoxia. Stem Cells Cloning.

[19] Sallam N.A., Palmgren V.A.C., Singh R.D., John C.M., Thompson J.A. (2018). Programming of vascular dysfunction in the intrauterine milieu of diabetic pregnancies. Int. J. Mol. Sci..

[20] Aboalgasm H., Ballo R., Gwanyanya A. (2021). Organisational alteration of cardiac myofilament proteins by hyperglycaemia in mouse embryonic stem cell-derived cardiomyocytes. J. Muscle Res. Cell Motil..

[21] Dluski D.F., Wolinska E., Skrzypczak M. (2021). Epigenetic changes in gestational diabetes mellitus. Int. J. Mol. Sci..

[22] Vrachnis N., Antonakopoulos N., Iliodromiti Z., Dafopoulos K., Siristatidis C., Pappa K.I., Deligeoroglou E., Vitoratos N. (2012). Impact of maternal diabetes on epigenetic modifications leading to diseases in the offspring. Exp. Diabetes Res..

[23] Slupecka-Ziemilska M., Wychowanski P., Puzianowska-Kuznicka M. (2020). Gestational diabetes mellitus affects offspring’s epigenome. Is there a way to reduce the negative consequences?. Nutrients.

[24] Gui J., Potthast A., Rohrbach A., Borns K., Das A.M., von Versen-Hoynck F. (2016). Gestational diabetes induces alterations of sirtuins in fetal endothelial cells. Pediatr. Res..

[25] Gui J., Rohrbach A., Borns K., Hillemanns P., Feng L., Hubel C.A., von Versen-Hoynck F. (2015). Vitamin d rescues dysfunction of fetal endothelial colony forming cells from individuals with gestational diabetes. Placenta.

[26] Blue E.K., Sheehan B.M., Nuss Z.V., Boyle F.A., Hocutt C.M., Gohn C.R., Varberg K.M., McClintick J.N., Haneline L.S. (2015). Epigenetic regulation of placenta-specific 8 contributes to altered function of endothelial colony-forming cells exposed to intrauterine gestational diabetes mellitus. Diabetes.

[27] Gohn C.R., Blue E.K., Sheehan B.M., Varberg K.M., Haneline L.S. (2017). Mesenchyme homeobox 2 enhances migration of endothelial colony forming cells exposed to intrauterine diabetes mellitus. J. Cell Physiol..

[28] Varberg K.M., Garretson R.O., Blue E.K., Chu C., Gohn C.R., Tu W., Haneline L.S. (2018). Transgelin induces dysfunction of fetal endothelial colony-forming cells from gestational diabetic pregnancies. Am. J. Physiol. Cell Physiol..

[29] Yadav S.S., Narayan G. (2014). Role of robo4 signalling in developmental and pathological angiogenesis. BioMed Res. Int..

[30] Li S., Huang L., Sun Y., Bai Y., Yang F., Yu W., Li F., Zhang Q., Wang B., Geng J.G. (2015). Slit2 promotes angiogenic activity via the robo1-vegfr2-erk1/2 pathway in both in vivo and in vitro studies. Investig. Ophthalmol. Vis. Sci..

[31] Liu J., Hou W., Guan T., Tang L., Zhu X., Li Y., Hou S., Zhang J., Chen H., Huang Y. (2018). Slit2/robo1 signaling is involved in angiogenesis of glomerular endothelial cells exposed to a diabetic-like environment. Angiogenesis.

[32] Aziz A., Haywood N.J., Cordell P.A., Smith J., Yuldasheva N.Y., Sengupta A., Ali N., Mercer B.N., Mughal R.S., Riches K. (2018). Insulinlike growth factor-binding protein-1 improves vascular endothelial repair in male mice in the setting of insulin resistance. Endocrinology.

[33] Slater T., Haywood N.J., Matthews C., Cheema H., Wheatcroft S.B. (2019). Insulin-like growth factor binding proteins and angiogenesis: From cancer to cardiovascular disease. Cytokine Growth Factor Rev..

[34] Fischer A., Schumacher N., Maier M., Sendtner M., Gessler M. (2004). The notch target genes hey1 and hey2 are required for embryonic vascular development. Genes Dev..

[35] Park J.K., Lee T.W., Do E.K., Moon H.J., Kim J.H. (2018). Role of notch1 in the arterial specification and angiogenic potential of mouse embryonic stem cell-derived endothelial cells. Stem Cell Res. Ther..

[36] Ying J., Li H., Seng T.J., Langford C., Srivastava G., Tsao S.W., Putti T., Murray P., Chan A.T., Tao Q. (2006). Functional epigenetics identifies a protocadherin *PCDH10* as a candidate tumor suppressor for nasopharyngeal, esophageal and multiple other carcinomas with frequent methylation. Oncogene.

[37] Xu Y., Yang Z., Yuan H., Li Z., Li Y., Liu Q., Chen J. (2015). *PCDH10* inhibits cell proliferation of multiple myeloma via the negative regulation of the wnt/beta-catenin/bcl-9 signaling pathway. Oncol. Rep..

[38] Shi D., Murty V.V., Gu W. (2015). *PCDH10*, a novel p53 transcriptional target in regulating cell migration. Cell Cycle.

[39] Houde A.A., Guay S.P., Desgagne V., Hivert M.F., Baillargeon J.P., St-Pierre J., Perron P., Gaudet D., Brisson D., Bouchard L. (2013). Adaptations of placental and cord blood abca1 DNA methylation profile to maternal metabolic status. Epigenetics.

[40] Dilli D., Dogan N.N., Ipek M.S., Cavus Y., Ceylaner S., Dogan H., Dursun A., Kucukozkan T., Zenciroglu A. (2018). Mafos-gdm trial: Maternal fish oil supplementation in women with gestational diabetes and cord blood DNA methylation at insulin like growth factor-1 (igf-1) gene. Clin. Nutr. ESPEN.

[41] Pauwels S., Ghosh M., Duca R.C., Bekaert B., Freson K., Huybrechts I., Langie S.A.S., Koppen G., Devlieger R., Godderis L. (2017). Maternal intake of methyl-group donors affects DNA methylation of metabolic genes in infants. Clin. Epigenetics.

[42] Ott R., Melchior K., Stupin J.H., Ziska T., Schellong K., Henrich W., Rancourt R.C., Plagemann A. (2019). Reduced insulin receptor expression and altered DNA methylation in fat tissues and blood of women with gdm and offspring. J. Clin. Endocrinol. Metab..

[43] Awamleh Z., Butcher D.T., Hanley A., Retnakaran R., Haertle L., Haaf T., Hamilton J., Weksberg R. (2021). Exposure to gestational diabetes mellitus (gdm) alters DNA methylation in placenta and fetal cord blood. Diabetes Res. Clin. Pract..

[44] Danese E., Minicozzi A.M., Benati M., Montagnana M., Paviati E., Salvagno G.L., Gusella M., Pasini F., Guidi G.C., Lippi G. (2013). Epigenetic alteration: New insights moving from tissue to plasma—The example of *PCDH10* promoter methylation in colorectal cancer. Br. J. Cancer.

[45] Liu W., Wu J., Shi G., Yue X., Liu D., Zhang Q. (2018). Aberrant promoter methylation of *PCDH10* as a potential diagnostic and prognostic biomarker for patients with breast cancer. Oncol. Lett..

[46] Seo S.I., Yoon J.H., Byun H.J., Lee S.K. (2021). Hotair induces methylation of *PCDH10*, a tumor suppressor gene, by regulating dnmt1 and sponging with mir-148b in gastric adenocarcinoma. Yonsei Med. J..

[47] Ying J., Gao Z., Li H., Srivastava G., Murray P.G., Goh H.K., Lim C.Y., Wang Y., Marafioti T., Mason D.Y. (2007). Frequent epigenetic silencing of protocadherin 10 by methylation in multiple haematologic malignancies. Br. J. Haematol..

[48] Park Y., Lee H.J., Jung Y.J., Kwon H.Y., Kim H., Lee J., Kim Y.H., Kim H.O., Maeng Y.S., Kwon J.Y. (2019). Cd133+/c-kit+lin(−) endothelial progenitor cells in fetal circulation demonstrate impaired differentiation potency in severe preeclampsia. Pregnancy Hypertens..

[49] Kwon H., Kwon J.-Y., Song J., Maeng Y.-S. (2021). Decreased lymphangiogenic activities and genes expression of cord blood lymphatic endothelial progenitor cells (vegfr3+/pod+/cd11b+ cells) in patient with preeclampsia. Int. J. Mol. Sci..

